# Mechanisms of Isoliquiritigenin Against Protein Glycation: A Comparative Study in PBS Solution and Crowding Environment

**DOI:** 10.3390/foods15101796

**Published:** 2026-05-19

**Authors:** Yushi Wei, Deming Gong, Guowen Zhang

**Affiliations:** State Key Laboratory of Food Science and Resources, Nanchang University, Nanchang 330047, China; weiyushi99@163.com (Y.W.); dgong01@gmail.com (D.G.)

**Keywords:** isoliquiritigenin, protein glycation, inhibitory mechanisms, macromolecular crowding

## Abstract

The advanced glycation end products generated from protein glycation are associated with the development of diabetic complications. This study aimed to investigate the inhibitory mechanisms of isoliquiritigenin on protein glycation and compare its anti-glycation activity in PBS versus a macromolecular crowding environment. The results showed that in PBS, 500 μmol/L isoliquiritigenin showed an advanced glycation end product inhibition rate of 37.78%, outperforming aminoguanidine. Meanwhile, isoliquiritigenin inhibited the protein carbonylation process, reduced the generation of protein oxidation products, and inhibited the formation of β-crosslinking structures with a rate of 34.20%. Molecular docking results indicated that isoliquiritigenin bound to site I of bovine serum albumin, effectively blocked glycation reactions by occupying multiple arginine residues and contributed to stabilizing the secondary structure of bovine serum albumin. In addition, isoliquiritigenin exhibited significant hydroxyl radical scavenging and Fe^2+^-chelating abilities, achieving a 34.35% trapping efficiency for methylglyoxal. Isoliquiritigenin exerted its anti-glycation activity through multiple pathways, including scavenging free radicals, protecting protein structure, interacting with bovine serum albumin, and trapping methylglyoxal. However, in the crowding environment, the excluded volume effect and higher viscosity might lead to limited isoliquiritigenin binding to bovine serum albumin, reducing its inhibition of glycation and decreasing advanced glycation end product inhibition to 16.38%. This study realistically evaluated the inhibitory effects of isoliquiritigenin in complex crowding environments and provided a theoretical basis for isoliquiritigenin as a functional food ingredient for the prevention of diabetes complications. Future studies need to establish animal models to further explore its effects in vivo.

## 1. Introduction

Hyperglycemia is a core driving factor in the development of diabetic complications, which promotes protein modification by inducing non-enzymatic glycation reactions, thereby facilitating the formation of advanced glycation end products (AGEs) [1]. In addition to endogenous pathological formation, exogenous dietary AGEs generated by the Maillard reaction during the heat processing of foods also serve as an important source of these products in the human circulatory system [2,3]. Accumulated AGEs promote the progression of diabetic complications through multiple mechanisms. Briefly, AGEs disrupt the normal structure, function and properties of proteins through covalent crosslinking, and AGEs activate inflammatory pathways, such as NF-κB, by binding to surface receptors, inducing oxidative stress and generating reactive oxygen species (ROS), which in turn accelerate the formation of AGEs [4].

Natural antioxidants, particularly polyphenolic compounds, have emerged as promising intervention strategies for inhibiting protein glycation and the formation of AGEs due to their high efficacy and safety [5]. Wei et al. [6] found that hesperidin inhibited the formation of AGEs and it effectively decreased the levels of soluble Aβ1-40 and ROS levels in the mouse hippocampus, thereby improving cognitive impairment. Zhao et al. [7] reported that four A-type proanthocyanidins could not only effectively reduce the formation of early Amadori products but also trap intermediate reactive carbonyl compounds; in addition, they protected bovine serum albumin (BSA) from glycation damage by reducing the binding of BSA to reducing sugars and blocking some glycation sites. Peng et al. [8] reported that the addition of a hesperetin–Cu(II) complex reduced the formation of AGEs in biscuits. Rosmarinic acid, resveratrol and epicatechin also significantly decreased the content of AGEs in cookies. Furthermore, resveratrol and epicatechin also reduced the levels of glyoxal and methylglyoxal [9]. In contrast, isoliquiritigenin (ISL), a natural chalcone derived from licorice and its related food products, has been widely reported to possess potential biological activities including anti-diabetic, antioxidant, immunomodulatory, anticancer, and the treatment of various inflammatory diseases [10,11]. However, its anti-glycation activity and the underlying molecular mechanisms remain largely unexplored. Notably, due to its physicochemical properties and metabolic losses, developing it as a dietary supplement could help increase its effective exposure level in the body, thereby enabling more stable biological activity.

More importantly, most current in vitro assessments of glycation inhibitors have been conducted in dilute solutions, which cannot truly reflect the complex intracellular environment. In fact, the cell interior is a highly crowded microenvironment where the concentration of biomacromolecules (such as proteins, nucleic acids, polysaccharides, etc.) can be as high as 80–400 mg/mL. This “macromolecular crowding environment” significantly influences intracellular biochemical reactions and molecular interactions [12]. The degree of molecular crowding is advocated as an important factor to describe environmental conditions in a solution (similar to pH, ionic strength, etc.). So far, several studies have revealed the impact of macromolecular crowding environments on the protein glycation process. Studies have shown that crowding environments triggered glycation reactions, and the extent of glycation increased with increasing concentrations of crowding agents, thereby shortening reaction times [13]. For example, macromolecular crowding environments enhanced the Maillard reaction between rice proteins and glucose, and the enhancement was related to the concentration of the crowding agent [14]. Perusko et al. [12] further found that crowded conditions not only enhanced the extent of glycation but also synergized with ultrasonication. Notably, the tertiary structure of whey protein did not significantly change due to the stabilizing effect of the crowding agent, thereby preventing excessive denaturation and aggregation of the whey protein. Similarly, Fuentes-Lemus et al. [15] reported that the glycation of plasma proteins was also modulated by crowding agents. Therefore, the inhibitory effects of anti-glycation agents observed in dilute solutions may not accurately reflect their true potential under physiologically relevant crowding conditions. However, studies on the anti-glycation activity of antioxidants, including ISL, under macromolecular crowding conditions are scarce, which greatly limits our understanding of their inhibitory efficacy in the authentic intracellular environment.

Therefore, in this study, a BSA–fructose model was established and multispectral techniques, inverted fluorescence microscopy, high-performance liquid chromatography (HPLC) and molecular docking were employed to systematically evaluate the inhibitory effect of ISL on protein glycation and its underlying mechanism in vitro. In contrast to previous studies that focused mainly on dilute solution conditions, this study simulated macromolecular crowding environment to build an in vitro evaluation system that more closely mimics physiological conditions, thereby allowing a more realistic assessment of the inhibitory effect of ISL in complex environments. This study provides a solid theoretical basis for the development of ISL as an efficient dietary supplement for the precise intervention of glycation-related diabetic complications.

## 2. Materials and Methods

### 2.1. Materials

BSA was purchased from Solarbio Science & Technology Co., Ltd. (Beijing, China). Fructose, ISL, methylglyoxal (MGO), polyethylene glycol 2000 (PEG), aminoguanidine hydrochloride (AG), nitrotetrazolium blue chloride (NBT), 2,4-dinitrophenylhydrazine (DNPH), thioflavin T (Tht) and FeCl_2_ were obtained from Aladdin Biochemical Technology Co., Ltd. (Shanghai, China).

### 2.2. Establishment of BSA–Fructose Model

The BSA–fructose glycation system was established based on the method of Peng et al. [8]. Briefly, BSA (20 mg/mL), fructose (90 mg/mL), and different concentrations of ISL (100, 300 and 500 μmol/L) or AG (500 μmol/L) were mixed in a PBS solution, pH 7.4, with or without PEG (100 g/L), then incubated at 50 °C and 100 r/min in the dark for 24 h.

### 2.3. Determination of Fluorescent AGEs

The diluted glycation sample (3 mL) was placed in a fluorescence cuvette. The fluorescence emission spectra were recorded from 400 nm to 600 nm at an excitation wavelength of 370 nm. The inhibition rate of ISL on fluorescent AGEs was calculated using the following equation:

*I* (%) = (*F*_0_ − *F_i_*)/*F*_0_ × 100 (1)

where *I* denotes the inhibition rate, and *F*_0_ and *F_i_* represent the fluorescence intensity of glycation samples without and with ISL treatment, respectively.

### 2.4. Protein Carbonylation Assay

The glycation sample was incubated with DNPH solution (200 µL) at 25 °C for 1 h. Then, 600 µL of trichloroacetic acid (20%, *w*/*v*) was added to precipitate the proteins, followed by incubation in an ice bath for 5 min and centrifugation at 10,000× *g* for 10 min. The supernatant was discarded, and the precipitate was washed with a mixture of ethanol and ethyl acetate (1:1, *v*/*v*). Finally, the precipitate was dissolved in guanidine hydrochloride solution (6 mol/L), and absorbance A of the sample at 360 nm was measured. The carbonylation content of the sample (*n*, µmol/g protein) was calculated according to the formula *n* = *A*/(mεl), where *n* is the concentration of BSA, and ε is the molar extinction coefficient, 2.2 × 10^4^ L/(mol·cm) [16].

### 2.5. Determination of Protein Thiol Content

The glycation sample was mixed with DTNB (200 μL, 2.0 mg/mL) and incubated at 25 °C for 20 min. The absorbance at 412 nm was measured, and the thiol content was calculated based on the Formula (2) [17]

C (μmol/g) = 73.53 × *A*_412nm_ × D/C_protein_ (2)

where C is the concentration of thiol groups, *A*_412nm_ is the absorbance at 412 nm, D is the dilution factor, and C_protein_ is the protein concentration.

### 2.6. Protein Oxidation Products

The detection of protein oxidation products was identified using fluorescence spectra. Excitation/emission wavelengths were set at 325/434 nm (N′-formylkynurenine), 330/415 nm (dityrosine) and 365/480 nm (kynurenine). The fluorescence intensity at each excitation wavelength of glycation samples was recorded to analyze the inhibitory effects of ISL on the formation of the three oxidation products [16].

### 2.7. Tht Measurement

The glycation sample (100 μL) was incubated with 50 μL of Tht solution (20 μg/mL) in the dark for 1 h. The excitation wavelength was set to 440 nm, and the fluorescence intensity at 500 nm was scanned to evaluate the effect of ISL on the protein β-crosslinking structure [3]. The fluorescence intensity of glycated BSA was set as 100%, and the percentage decrease in fluorescence at 500 nm was defined as the effect of ISL/AG on the protein β-crosslinking structure. The cross-β structures of proteins in the Tht-stained mixture were observed using an IX3-RFACS inverted fluorescence microscope (Olympus, Tokyo, Japan), and quantitative analysis was performed using ImageJ software (version 1.54g, Bethesda, MD, USA).

### 2.8. Fluorescence-Quenching Assay

The ISL solution (0.26 mg/mL) was added dropwise into 2 mL of BSA (0.2 mg/mL) in the PBS or PEG system. The mixture was incubated for 2 min for equilibration before the measurements. Fluorescence emission spectra of samples were recorded using a fluorescence spectrophotometer (F-7100, Hitachi, Tokyo, Japan) with an excitation wavelength of 280 nm, and the measured data were corrected. The fluorescence-quenching constant (*K*_sv_), quenching rate constant (*K*_q_), binding constant (*K*_a_), and number of binding sites (*n*) were calculated, based on the Stern–Volmer Equation (3) and the double logarithmic Equation (4), respectively.

(3) $$\frac{F_{0}}{F}=K_{\mathrm{SV}}[I]+1=K_{q}\tau_{0}[I]$$

(4) $$\log\frac{F_{0}-F}{F}=n\log K_{a}-n\log\frac{1}{\left[I]-\frac{\left(F_{0}-F\right)}{F_{0}}\cdot [Q\right]}$$

where *F*_0_ and *F* represent the fluorescence intensity of BSA and ISL-treated BSA, respectively. [*I*] is the concentration of ISL. τ_0_ represents the fluorescence lifetime of the enzymes, which is usually 10^−8^ s. [*Q*] is the concentration of BSA.

### 2.9. Circular Dichroism (CD) Spectra

Native BSA solution was diluted to 0.5 mg/mL, and the glycation sample was diluted accordingly. After subtracting the solvent background, CD spectra were measured, and the protein secondary structure content was calculated [18].

### 2.10. Molecular Docking

Molecular docking between ISL and BSA was performed using the AutoDockTools 1.5.7 software. The crystal structure of BSA (PDB ID: 4F5S) was obtained from the RCSB Protein Data Bank, and the three-dimensional structure of ISL was retrieved from the PubChem database. Before docking, BSA was prepared by removing water molecules and any irrelevant ligands, and polar hydrogens were added. Blind docking was performed, with the entire BSA protein designated as the docking region. The cubic grid box was installed at 108 Å × 94 Å × 120 Å with 0.581 Å apart, and the number of genetic algorithms was set at 100. The conformation exhibiting the lowest binding energy between ISL and BSA was selected from the docking results, and was subsequently visualized and analyzed using Discovery Studio Visualizer (version 2019) [19].

### 2.11. Determination of Free Radical Scavenging Activity

In PBS or PEG solution, different concentrations of ISL were mixed with FeSO_4_ (1 mmol/L), sodium benzoate (2 mM) and ethylenediaminetetraacetic acid (1 mM). Subsequently, H_2_O_2_ (2 mmol/L) was added, and the mixture was incubated at 37 °C for 2 h, with the vitamin c (Vc) group serving as a positive control. The excitation wavelength was set at 305 nm, and the fluorescence intensity of samples at 418 nm was measured. The scavenging rate of hydroxyl radical was calculated using the Formula (5) [20].

The scavenging rate (%) = [1 − (*F*_2_ − *F*_0_)/(*F*_1_ − *F*_0_)] × 100 (5)

where *F*_2_ and *F*_1_ are the fluorescence intensity of the reaction system in the presence or absence of ISL or ascorbic acid, respectively. *F*_0_ is the fluorescence intensity of PBS solution with or without PEG.

### 2.12. Determination of Metal Ion Chelating Capacity

A series of concentrations of ISL/ehylenediaminetetraacetic acid (EDTA)/AG were incubated with 200 μL of FeCl_2_ solution at 25 °C for 10 min in PBS or PEG solution. Subsequently, 200 μL Ferrozine (5 mmol/L) was added, and the mixture was shaken for 10 min. The absorbance of the samples was measured at 562 nm and the chelating rate of ISL for Fe^2+^ was calculated based on the Formula (6) [16].

Fe^2+^ chelation rate (%) = (*A*_0_ − *A_i_*)/*A*_0_ × 100 (6)

where *A*_0_ and *A_i_* are the absorbance of the reaction system in the absence or presence of ISL respectively.

### 2.13. MGO-Trapping Capability

The MGO solution was mixed with different concentrations of ISL (300, 500 and 1000 μmol/L) in PBS (pH 7.4), and incubated at 37 °C with shaking for 24 h. After the reaction, acetic acid was immediately added to quench the reaction, followed by the addition of o-phenylenediamine and 5-methylquinoxaline, and the reaction was allowed to continue for 30 min. The formed derivative 2-methylquinoxaline (2-MQ) was detected by HPLC to quantitatively analyze the trapping rate of ISL on MGO. In addition, to investigate the time kinetics of ISL (1000 μmol/L) trapping MGO, the sample was incubated at 37 °C with shaking according to the above method, and then it was taken at 0.5, 1, 2, 4, 6, 12, and 24 h to determine the residual MGO content.

HPLC analysis was performed on an Agilent ZORBAX Eclipse XDB-C18 column (5 μm, 4.6 mm × 250 mm, Agilent, Santa Clara, CA, USA), with an injection volume of 10 μL and a column temperature of 30 °C. Detection wavelength was set at 317 nm. Mobile phase A was 0.1% formic acid in water and mobile phase B was 100% methanol. An isocratic elution program (50% B) was applied at a flow rate of 1 mL/min for 20 min.

### 2.14. Statistical Analysis

Three parallel samples were performed for all experiments and the results were expressed as mean ± standard deviation (*n* = 3). The data were statistically analyzed through one-way analysis of variance (ANOVA) using IBM SPSS statistical software version 19 (SPSS Inc., Chicago, IL, USA). Statistical significance was defined as *p* < 0.05.

## 3. Results and Discussion

### 3.1. Inhibition of AGEs

**Figure 1:**
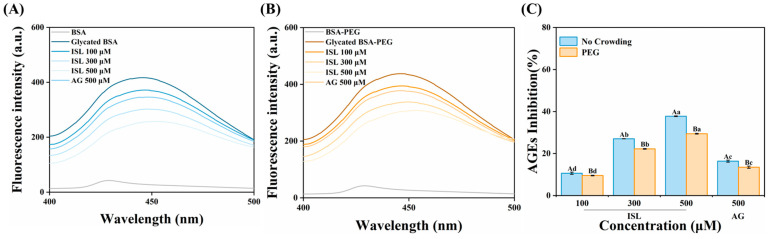
Fluorescence spectra (λ_ex_ = 370 nm) of AGEs in glycated BSA treated with ISL (100, 300 and 500 μmol/L)/AG (500 μmol/L) in the absence (**A**) or presence of PEG (**B**) after incubation at 50 °C for 24 h (pH 7.4). Inhibition rates of AGEs (**C**) by ISL/AG without and with PEG. Different uppercase and lowercase letters indicate significant differences within and among groups, respectively (*p* < 0.05). Abbreviations: AGEs, advanced glycation end products; BSA, bovine serum albumin; ISL, isoliquiritigenin; AG, aminoguanidine hydrochloride; PEG, polyethylene glycol.

The formation and accumulation of fluorescent AGEs serve as key targets for evaluating anti-glycation activity, and inhibiting their generation is of great significance. Fluorescent AGEs can be evaluated by measuring the fluorescence intensity around 450 nm. As the concentration of ISL increased, the fluorescence intensity of AGEs significantly decreased, with the highest inhibition rate reaching 37.78% (Figure 1C). It demonstrated that ISL had a stronger inhibitory effect on AGEs than AG, highlighting its potential as a natural anti-glycation agent. Previous research has shown that the antioxidant capacity of compounds was crucial for their anti-glycation activity, and the antioxidant capacity of polyphenolic compounds was closely related to the number of hydroxyl groups they possessed [21]. For example, Anis & Sreerama [22] reported that the free radical scavenging abilities of p-coumaric acid, ferulic acid, vanillic acid, and gallic acid during the glycation process were associated with the inhibition of AGE formation. Peng et al. [8] also reported that hesperetin exhibited a superior inhibitory effect on AGEs due to its strong antioxidant capacity compared to AG. However, the inhibitory effect of ISL on glycation products appeared to be less potent than other phenolic compounds with a similar number of hydroxyl groups, which may also be related to its structure. For example, resveratrol also contained three hydroxyl groups [23]; its smaller dihedral angle and the closer coplanarity of rings A and B enlarged the π-conjugated system, generating a more stable conjugated structure, which enhanced planarity, facilitating electron delocalization and radical stabilization, thereby conferring greater antioxidant activity [24]. Notably, PEG alone could slightly promote the formation of AGEs, indicating that crowding agents could promote the development of glycation [12]. Zhang et al. [20] also reported similar results. Compared to PBS, the presence of PEG weakened the inhibitory effect of ISL and AG, which may be because PEG increased the viscosity of the system, thereby affecting the binding of the inhibitor to glycated BSA [15]. Nevertheless, the inhibitory effect of ISL remained stronger than that of AG under the same conditions.

### 3.2. Inhibition of Protein Carbonylation

**Figure 2:**
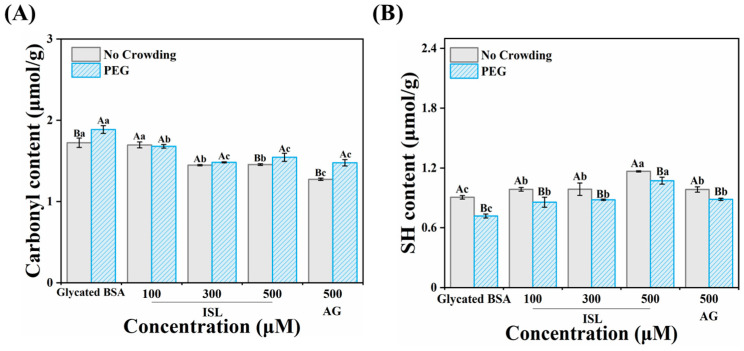
Effects of ISL (100, 300 and 500 μmol/L)/AG (500 μmol/L) on protein carbonylation (**A**) and protein thiol content (**B**) in glycated BSA in the presence or absence of PEG after incubation at 50 °C for 24 h. Different uppercase and lowercase letters indicate significant differences within and among groups, respectively (*p* < 0.05). Abbreviations, BSA, bovine serum albumin; ISL, isoliquiritigenin; AG, aminoguanidine hydrochloride; PEG, polyethylene glycol.

During glycation, reducing sugars undergo autoxidation or oxidative degradation of Amadori intermediates to form reactive carbonyl intermediates (RCIs). These RCIs interact with cysteine and histidine residues or directly oxidize arginine, lysine and tyrosine residues, resulting in the formation of protein carbonyl derivatives. In addition, the reactive oxygen species (ROS) generated during this process also oxidize the side chains of amino acid residues in the protein, further forming carbonyl derivatives [25]. As shown in Figure 2A, exposure of BSA to fructose significantly increased protein carbonyl content, reaching 1.72 μmol/g. Treatment with different concentrations of ISL (100, 300 and 500 μmol/L) reduced the carbonyl content of the glycated protein to 1.70, 1.45 and 1.46 μmol/g, respectively. The positive control, AG (500 μmol/L), also decreased the carbonyl content in glycated BSA to 1.27 μmol/g. Studies have shown that non-enzymatic reactions and free radical-induced protein oxidation led to the formation of AGEs and protein damage [26]. Considering that free radicals can oxidize amino acid residues on protein side chains, the reduction in carbonyl content in glycated BSA treated with ISL may be related to its antioxidant activity in scavenging free radicals and ROS. Ma et al. [27] also reported that the isolated polysaccharide FJP-1 exhibited strong inhibition of protein carbonyl formation through scavenging of free radicals and reactive oxygen species, thereby preventing the generation of carbonyl groups. In addition, PEG elevated the carbonyl content of glycated BSA to 1.89 μmol/g, which may be attributed to the excluded volume effect generated by crowding, which increased the local effective concentration of proteins and glycating agents, enhanced the frequency of reactive collisions, and accelerated the glycation process. Meanwhile, the macromolecular crowding environment increased the degree of protein oxidative modification [28]. Compared with PBS, the inhibitory effect of ISL on the carbonylation of glycated BSA in PEG solution was slightly weakened, presumably because the crowding agent modulated the process of carbonyl accumulation [6]. In summary, ISL effectively inhibited the development of BSA carbonylation in different solution environments.

### 3.3. Protective Effect on Protein Thiols

Cysteine residues in proteins are susceptible to oxidation, leading to the formation of disulfide bonds and protein aggregation. Therefore, thiol content is considered as an important indicator for the extent of protein oxidation during glycation. As shown in Figure 2B, compared to native BSA (2.03 μmol/g), the thiol content of fructose-mediated glycated BSA decreased to 0.91 μmol/g, indicating that part of the BSA thiols were consumed during the protein glycation process [29]. Both the positive control AG (500 μmol/L, 0.985 μmol/g) and ISL exhibited certain activity in restoring free thiol groups. After treatment with ISL at 100, 300 and 500 μmol/L, the thiol contents were increased to 0.986, 0.988 and 1.17 μmol/g, respectively, indicating that ISL was more effective than AG in reversing BSA thiol depletion. In addition, the protective effect of ISL on protein thiols was reduced in the presence of PEG; after treatment with 500 μmol/L ISL, the thiol content of glycated BSA was 1.07 μmol/g, but its protective ability was still higher than that of AG. The antioxidant activity of phenolic compounds was closely related to their ability to inhibit the oxidation of protein residues. However, ISL exhibited weaker protection of thiols compared to polyhydroxy flavonoids such as kaempferol and quercetin. This may be because the flavonoids with four or six hydroxyl groups in their structure can more effectively protect thiols in proteins from oxidation [22]. Deng et al. [26] reported that the presence of transition metal ions can accelerate the consumption of free thiols in proteins, consistent with the results in Section 3.10 showing that ISL can effectively chelate metal ions.

### 3.4. Inhibition of Protein Oxidation Products

**Figure 3:**
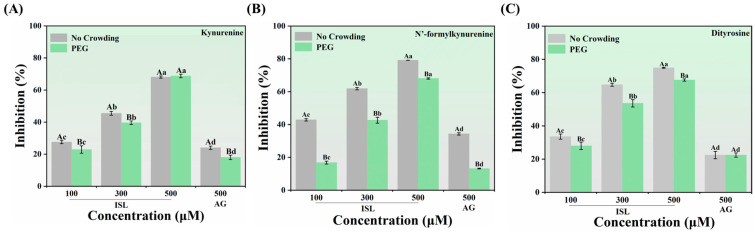
Inhibition rates of kynurenine (**A**), N′-formylkynurenine (**B**) and dityrosine (**C**) by ISL (100, 300 and 500 μmol/L)/AG (500 μmol/L) in the presence or absence of PEG. Different uppercase and lowercase letters indicate significant differences within and among groups, respectively (*p* < 0.05). Abbreviations: ISL, isoliquiritigenin; AG, aminoguanidine hydrochloride; PEG, polyethylene glycol.

Kynurenine, N′-formylkynurenine and dityrosine are the products of protein oxidation (tyrosine and tryptophan residues) during protein glycation. The level of these oxidation products reflects the extent of protein oxidation induced by glycation. ISL exhibited a dose-dependent inhibitory effect on the formation of kynurenine, N′-formylkynurenine and dityrosine (Figure 3A–C). When the concentration of ISL reached 500 μmol/L, the fluorescence intensities of kynurenine, N′-formylkynurenine and dityrosine were suppressed by 68.06%, 79.17% and 74.91%, respectively, which was better than the suppression achieved with 500 μmol/L AG (24.01%, 34.29% and 22.43%). Notably, the addition of PEG alone promoted the formation of protein oxidation products. Under macromolecular crowding conditions, the inhibitory effect of ISL was reduced. This may be due to the excluded volume effect caused by the crowding agent PEG, which weakened the protective effect of ISL on protein amino acid residues. Similar results were obtained by Zhang et al. [20]. Additionally, the inhibitory effect of ISL on kynurenine was weaker than N′-formylkynurenine and dityrosine. This was because kynurenine and N′-formylkynurenine/dityrosine were oxidation products originating from different protein amino acid residues, and ISL provided more significant protection for tryptophan residues in BSA [7]. Similarly, Xu et al. [30] reported that betanin showed different degrees of inhibition against the oxidation of two amino acid residues, with a more pronounced suppression of kynurenine. These results demonstrated that ISL effectively protected the tryptophan and tyrosine residues of BSA from glycoxidation, thereby reducing the risk of protein damage.

### 3.5. Inhibition of Protein Crosslinking

**Figure 4:**
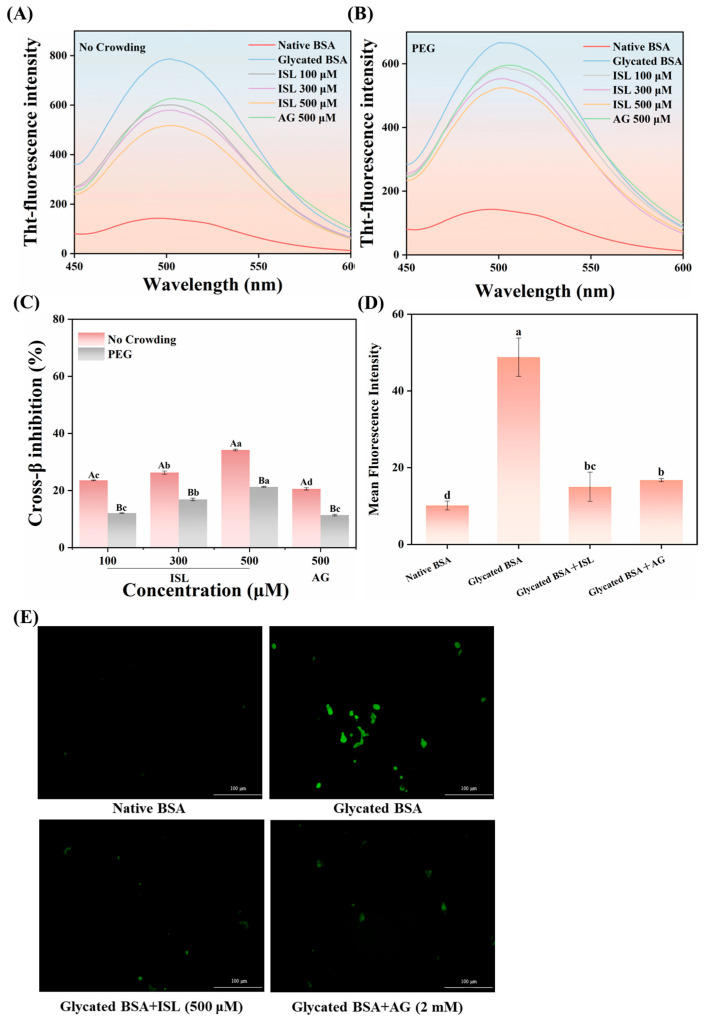
Effects of ISL/AG on the fluorescence intensity of glycated BSA with Tht in the absence (**A**) or presence (**B**) of PEG in the dark for 1 h (λ_ex_ = 440 nm). (**C**) The inhibitory activity of ISL/AG against cross-β structures. (**D**) Quantitative analysis of cross-β structure fluorescence intensity. (**E**) Inverted fluorescence images of Tht-stained glycated BSA. Different uppercase and lowercase letters indicate significant differences within and among groups, respectively (*p* < 0.05). Abbreviations: Tht, thioflavin T; ISL, isoliquiritigenin; BSA, bovine serum albumin; AG, aminoguanidine hydrochloride; PEG, polyethylene glycol.

Advanced glycation can induce protein crosslinking, aggregation and conformational changes, leading to the transformation of proteins from the native structure to non-native conformations enriched with β-sheets, including β-amyloid cross-structures, and exacerbating amyloid protein aggregation. The accumulation of these β-amyloid cross-structures in specific organs or tissues can lead to amyloidosis, which plays a crucial role in the progression of type II diabetes and neurodegenerative diseases [31]. When Tht bound to β-amyloid cross-structures, a significant fluorescence peak was observed at a wavelength of 500 nm (Figure 4A,B). Interestingly, it was found that the fluorescence intensity of glycated BSA in a PEG environment was lower than that in a PBS environment. This result was consistent with Zhang et al. [32] showing that crowding agents prevented the excessive aggregation of β-conglycinin. Regardless of whether in the PEG or PBS environment, ISL treatment could reduce the Tht fluorescence intensity of glycated BSA. In the PBS environment, the inhibition rates of ISL at 100, 300 and 500 μmol/L against the β-amyloid crosslinking structure were 23.57%, 26.29% and 34.20%, respectively (Figure 4C). In the PEG environment, although the inhibitory efficacy of ISL was reduced, the inhibition rates at the same concentrations remained at 12.19%, 16.93%, and 21.31%, respectively, and were all higher than those of AG. These results indicated that ISL effectively inhibited the formation of amyloid fibril aggregates in glycated BSA, thus exerting an anti-protein glycation effect.

Tht, as a specific fluorescent dye, can generate fluorescent signals by binding to protein aggregates, enabling the visualization of protein aggregates through inverted fluorescence microscopy. As shown in Figure 4D, nearly no green fluorescent spots were observed in native BSA, indicating the absence of amyloid cross-structures. However, large and elongated green fluorescent plaques were visible in glycated BSA, confirming that the glycation process induced partial crosslinking of the protein, leading to the formation of amyloid aggregates. After treatment with 500 μmol/L ISL, the size of the green fluorescent plaques and aggregates decreased significantly, contrasting sharply with the long-chain green amyloid aggregates observed in untreated glycated BSA. Notably, dark green block-like structures were still evident in AG-treated glycated BSA, while the number and size of green plaques in ISL-treated samples were reduced further (Figure 4D), indicating that ISL was more effective than AG in inhibiting β-amyloid crosslinking structures. Liu et al. [5] observed a reduction in protein aggregates after the addition of natural antioxidants to the BSA-MGO system.

### 3.6. Interaction Between ISL and BSA

**Figure 5:**
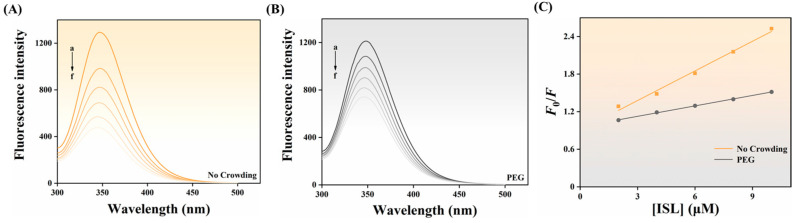
Fluorescence-quenching spectra (λ_ex_ = 280 nm) of BSA (0.2 mg/mL) with ISL (**A**,**B**) (a → f: 0, 2, 4, 6, 8, 10 μmol/L) and Stern–Volmer plots (**C**) in the absence and presence of PEG. Abbreviations: BSA, bovine serum albumin; ISL, isoliquiritigenin; AG, aminoguanidine hydrochloride; PEG, polyethylene glycol.

Phenolic compounds can protect glycosylation sites on proteins by forming stable complexes with proteins, which is an important mechanism by which they exert their anti-glycosylation activity [33]. Fluorescence quenching was employed to characterize the binding between proteins and phenolic compounds, and the interaction between ISL and BSA was investigated to elucidate the molecular mechanism by which ISL inhibited BSA glycation. In PBS or PEG systems, BSA exhibited a distinct fluorescence emission peak at around 346 nm when excited at a wavelength of 280 nm. As the concentration of ISL increased, the fluorescence intensity of BSA gradually decreased in a dose-dependent manner (Figure 5A–C). This was primarily attributed to the formation of non-fluorescent substrate complexes between aromatic amino acids and ISL, thereby inducing BSA fluorescence quenching [34]. Fluorescence-quenching mechanisms are usually categorized as static quenching, dynamic quenching, or a combination of both, with the Stern–Volmer plot used to identify the potential quenching type. As shown in Figure 5C, the Stern–Volmer plot of *F*_0_/*F* versus ISL concentration exhibited a good linear relationship, indicating that the quenching process between ISL and BSA was either a single static or dynamic quenching. The calculated *K*_q_ values for the PBS and PEG systems were 1.45 and 0.50 × 10^13^ L mol^−1^ s^−1^, respectively (Table 1). These values were significantly higher than the maximum diffusion collision quenching constant, indicating that the quenching mechanism of ISL on BSA was static quenching in both systems, and ISL formed a complex with BSA [35]. In addition, the *K*_sv_ value in the PEG system was significantly smaller than that in the PBS system, indicating that the macromolecular crowding environment reduced the efficiency of fluorescence quenching of BSA by ISL. Based on the double logarithm equation, the *K*_a_ value and *n* between ISL and BSA were further calculated (Table 1). In both systems, the *n* value was approximately one, indicating that there was only one binding site for ISL on BSA. In PBS, the *K*_a_ value was 18.89 × 10^4^ L/mol, which was at the 10^5^ order of magnitude, indicating strong interaction between ISL and BSA. The *K*_a_ value in the PEG system was lower than that in PBS, implying that the macromolecular crowding environment reduced the binding affinity between ISL and BSA. These results suggested that the strong interaction between ISL and BSA can hinder the binding of fructose to BSA, thereby effectively inhibiting the glycation process [7]. Furthermore, crowding conditions interfered with the interaction between BSA and ISL, thereby reducing the ability of ISL to inhibit the non-enzymatic glycation of BSA. This effect can be attributed to the excluded volume effect induced by PEG, which limited molecular motion and the probability of effective collisions. Additionally, an increase in system viscosity hindered the diffusion and binding of ISL to the active site of BSA. These factors collectively influenced intermolecular binding behavior [36].

### 3.7. Protective Effect on the Secondary Structure of BSA

**Figure 6:**
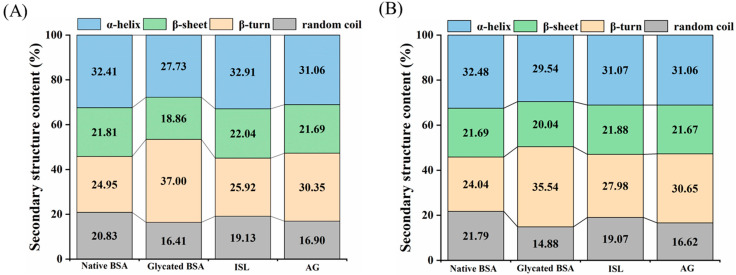
Effects of ISL (**A**)/AG (**B**) on the secondary structure of BSA (0.5 mg/mL) in the presence or absence of PEG. Abbreviations: BSA, bovine serum albumin; ISL, isoliquiritigenin; AG, aminoguanidine hydrochloride; PEG, polyethylene glycol.

Native BSA exhibited negative absorption peaks at 208 and 220 nm, indicating the presence of α-helices and β-sheets, which were characteristic features of protein structures. After glycation, the α-helix contents of BSA were decreased by 4.42% and 6.91% in PBS and PEG environments, respectively (Figure 6A,B). This reduction was attributed to the binding of the protein to fructose molecules, leading to the unfolding of the protein and exposure of internal groups, thus disrupting the stability of the α-helix structure [37]. Meanwhile, the β-sheet contents were increased by 12.05% and 11.50%, respectively, which was the intrinsic driving force for the formation of glycation aggregates. Fructose-induced BSA glycation altered the secondary structure of BSA by disrupting intramolecular interactions [38]. Notably, the increase in β-sheet content was lower in the PEG environment compared to the PBS environment, consistent with a previous study showing that crowding agents inhibited excessive protein aggregation [32]. After ISL/AG treatment, the contents of α-helix and β-sheet in glycated BSA exhibited varying degrees of recovery. In PBS solution, the α-helix content increased from 16.41% to 19.13%/16.90%, while the β-sheet content decreased from 37.00% to 25.92%/30.35%. These results indicated that ISL/AG was able to resist changes in the secondary structure of glycated BSA, thereby mitigating the aggregation of glycated BSA. Zhu et al. [38] reported that catechins significantly restored the β-sheet structure of glycated BSA and inhibited the aggregation of large molecules. Wang et al. [39] also reported that four polyphenols increased the content of lactoferrin α-helices when combined with lactoferrin, thereby stabilizing the structure of lactoferrin. However, the protective effect of ISL/AG in crowded environments was weaker than in PBS solution, which was due to PEG-induced perturbations in the secondary structure of BSA and its impact on the binding between ISL and BSA. These results were consistent with a previous study showing that crowding agents diminished the protective effect of chicoric acid on the secondary structure of BSA [20].

### 3.8. Molecular Docking Analysis

**Figure 7:**
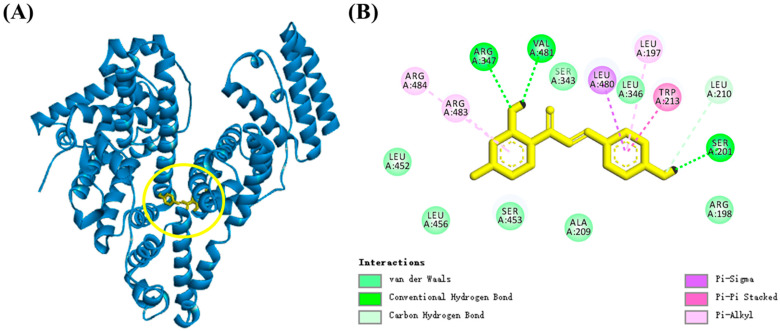
(**A**) The diagrams of molecular docking between BSA and ISL (the yellow circle indicated highlighting). (**B**) 2D schematic of interaction type of BSA with ISL. Abbreviations: BSA, bovine serum albumin; ISL, isoliquiritigenin.

Molecular docking can provide a more intuitive understanding of intermolecular binding characteristics. Molecular docking techniques were utilized to further elucidate the interaction between ISL and BSA and determine the binding site. The results of molecular docking showed that ISL bound with a binding energy of (−31.68 kJ mol^−1^) to subdomain IIA (Sudlow’s site I) of BSA [33] (Figure 7). ISL was surrounded by many hydrophobic amino acid residues (Leu, Val, Ala and Trp) and hydrophilic amino acid residues (Arg and Ser). ISL formed three hydrogen bonds with Arg347, Val481 and Ser201, with bond lengths of 2.2 Å, 2.2 Å, and 1.9 Å, respectively. These hydrogen bonds enhanced the binding affinity between ISL and BSA. Several π interactions were present between the benzene ring of ISL and Leu197, Trp213, Leu480, Arg483 and Arg484. Specifically, ISL formed a π-π stacking interaction with Trp213, a hydrophobic interaction which can reasonably account for the fluorescence quenching of the protein observed upon ISL binding [40]. Concurrently, ISL also established pi-alkyl interactions with Leu197, Arg483, and Arg484, and a pi-sigma interaction with the Leu480 residue. In addition, ISL interacted with Arg198, Ala209, Ser343, Leu346, Leu452, Ser453, and Leu456 by van der Waals forces. These intermolecular forces enhanced the binding affinity of ISL to BSA and contributed to the stabilization of the spatial structure of BSA. It was reported that arginine (Arg), lysine (Lys), and cysteine (Cys) were the primary sites for glycation modification of BSA due to their strong nucleophilicity. Among these, Lys and Arg were also key precursors for pentosidine [41]. These results demonstrated that ISL can block multiple ARG sites in BSA, including Arg198, Arg347, Arg483 and Arg484, and ISL effectively inhibited glycation modification and protein aggregation by occupying arginine residues and stabilizing the BSA structure, thereby reducing the formation of glycated products [30]. These results not only provided further support for the results presented in Section 3.1, but also deepened the understanding of the molecular mechanisms by which ISL mitigated the glycation process.

### 3.9. Free Radical Scavenging Ability

**Figure 8:**
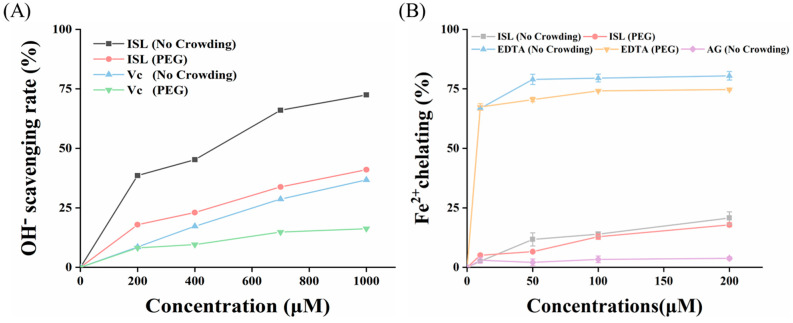
Fe^2+^-chelating activity (**A**) and free radical scavenging ability (**B**) of ISL and Vc/EDTA in the presence or absence of PEG. Abbreviations: ISL, isoliquiritigenin; AG, aminoguanidine hydrochloride; PEG, polyethylene glycol; Vc, vitamin c; EDTA, ehylenediaminetetraacetic acid.

Hydroxyl radicals are one of the most reactive free radicals among ROS. They can attack specific amino acid residues within proteins, leading to oxidative modifications such as protein carbonylation, which in turn can trigger oxidative stress. Therefore, scavenging hydroxyl free radicals to reduce oxidative stress and decrease the formation of active carbonyl or dicarbonyl compounds can effectively inhibit the progression of glycation. As shown in Figure 8A, in PBS solution, the ability of ISL to scavenge hydroxyl radicals significantly increased with increasing concentration. Specifically, the scavenging rates reached 38.61%, 45.25%, 66.05% and 72.46% at ISL concentrations of 200, 400, 700 and 1000 μmol/L, respectively. The free radical scavenging ability of ISL was attributed to its phenolic hydroxyl structure, which is also related to inhibiting the generation of free radicals through metal ion chelation, thus exhibiting strong hydroxyl radical scavenging activity [27]. In addition, the antioxidant capacity of flavonoids is closely related to their molecular structure. The C-ring pyranone structure (containing a C-2,3 double bond and a C-4 carbonyl group) of flavonoids, such as vitexin, formed a conjugated system with the phenolic hydroxyl groups on the A and B rings [16]. This conjugation effect stabilized free radical intermediates, thereby enhancing antioxidant ability. In contrast, the open C-ring structure of ISL lacked this conjugated system, resulting in poorer free radical stability and thereby somewhat weakening its antioxidant capacity [42]. Notably, ISL exhibited weaker scavenging activity in PEG solution than in PBS, only inhibiting 17.90%, 23.03%, 33.79%, and 41.06% at the same concentration. This was due to the excluded volume effect induced by PEG, where in crowded environments, protein molecules entered a state of higher local concentration, affecting the diffusion and reaction kinetics of protein molecules and ISL [43]. Nevertheless, ISL also exhibited significantly superior scavenging ability compared to the positive control Vc, and the free radical scavenging ability of ISL was crucial for inhibiting AGE formation.

### 3.10. Chelating Ability of Metal Ions

Fe^2+^ can catalyze the decomposition of H202 to generate highly reactive hydroxyl radicals. Due to its extreme reactivity, Fe^2+^ is considered a strong pro-oxidant ion. Therefore, chelating Fe^2+^ may be an effective strategy for preventing diseases induced by oxidative stress. As shown in Figure 8B, ISL effectively chelated Fe^2+^ in a concentration-dependent manner; in a PBS environment, 200 μmol/L ISL chelated nearly 25% of Fe^2+^. Although weaker than the strong metal-chelating ability of EDTA, ISL still demonstrated effective chelating capability and the chelating capacity of ISL did not change significantly in the PEG environment. ISL chelated metal ions through adjacent hydroxyl and carbonyl functional groups in the molecule. Similarly, flavonoids, such as hesperetin, also chelated metal ions (Cu^2+^) through functional carbonyl groups [8]. Additionally, two ISL molecules also could chelate metal ions through the meta-hydroxyl groups on A-rings. Hydroxyl functional groups were reportedly capable of exhibiting metal-chelating activity when in an appropriate structure–function configuration, as exemplified by resveratrol chelating metal ions in the similar manner [44]. ISL, as a chalcone, lacks C-ring and the C-ring double bond, which somewhat reduced its metal-chelating ability, yet it still demonstrated effectiveness. In contrast, some flavonoids (such as quercetin) possessed C-ring and the C-ring double bond that enhanced molecular rigidity and maintained higher planarity between the A and C rings. It made the adjacent hydroxyl and carbonyl groups closer, further improving the chelating ability [45]. Transition metal ions can act as mediators in the glycation process, leading to the formation of AGEs. The ability of ISL to chelate metal ions may be correlated with its activity in inhibiting AGE formation [46], consistent with the results in Section 3.1. It further supported the potential of ISL to exert its antioxidant and anti-glycation effects by chelating metal ions.

### 3.11. MGO-Trapping Ability

**Figure 9:**
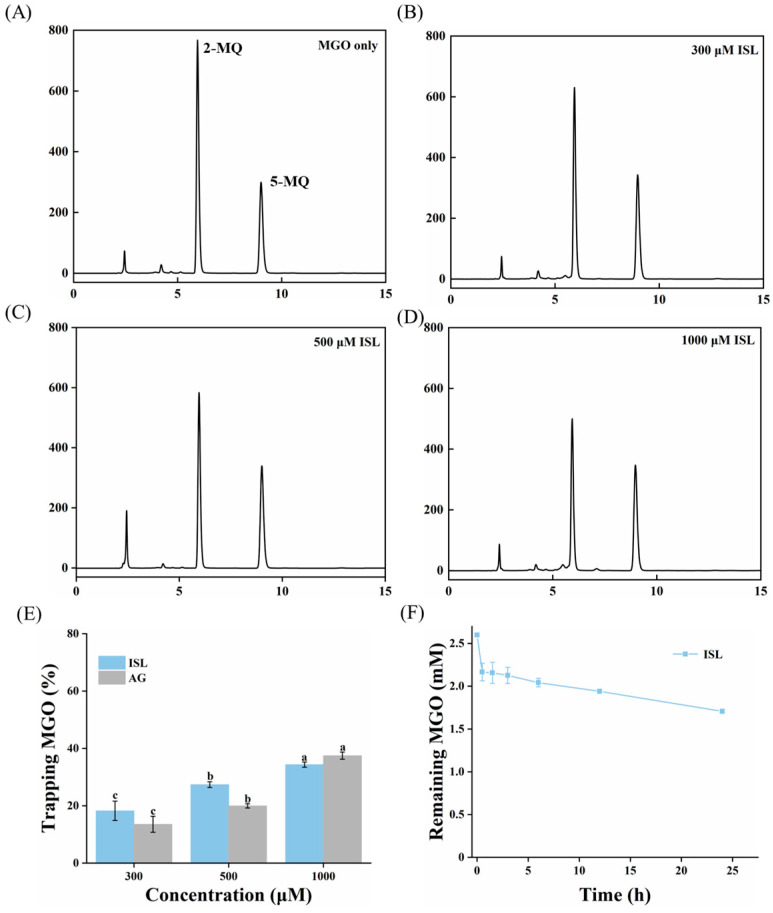
(**A**–**D**) HPLC chromatogram of MGO after trapping with ISL (0, 300, 500 and 1000 μmol/L); trapping rate of MGO by ISL (300, 500 and 1000 μmol/L); (**E**) trapping dynamics of MGO by ISL (1000 μmol/L) within 24 h. Different lowercase letters indicate significant differences among groups (*p* < 0.05); (**F**) MGO was incubated with ISL in PBS (pH 7.4) at 37 °C for 24 h. Abbreviations: ISL, isoliquiritigenin; MGO, methylglyoxal.

MGO is a highly reactive dicarbonyl compound that readily attacks specific amino acids in proteins (such as lysine and arginine) to produce AGEs. It is one of the key precursor substances for AGE formation. Therefore, trapping MGO to block its reaction with proteins is an effective strategy to inhibit AGE formation. HPLC quantification of the trapping MGO efficiency of ISL revealed that the amount of 2-MQ formed from MGO and o-phenylenediamine decreased progressively with increasing ISL/AG concentration. This indicated that ISL/AG effectively reduced the residual MGO content in the system (Figure 9A–D). After treatment with 1000 μL ISL/AG, the MGO-trapping efficiency reached 34.35% and 37.49%, respectively (Figure 9E). The MGO-trapping efficiency of low-concentration ISL was higher than that of AG, while that of high-concentration ISL was lower than that of AG. This may be related to the number of binding sites available for MGO capture in a trapping agent. Peng et al. [8] reported that hesperetin exhibited significantly higher MGO-trapping efficiency compared to AG, due to its active sites on the compounds involved in trapping MGO. In addition, this study further investigated the MGO-trapping efficiency of ISL over 24 h and trapping kinetic characteristics. The kinetic curves revealed that the MGO-trapping rate by ISL exhibited a rapid initial phase, followed by a gradual slowing (Figure 9F), indicating a faster initial capture rate, then a gradual plateau. Wu et al. [47] also reported a similar kinetic trend of catechin in trapping MGO. Structurally, when the flavonoid A-ring possessed hydroxyl substituents at the C-5 or C-7 positions, C-6 or C-8 on the A-ring served as the primary active site for trapping reactive dicarbonyl compounds and forming adducts. Furthermore, the hydroxyl group at the C-5 position on the A-ring, due to its electron-donating properties under slightly alkaline conditions, could significantly enhance the dicarbonyl compound trapping capacity of flavonoid. ISL possessed the structural features required for efficient MGO trapping; thus, MGO might bind to the C-6 or C-8 active sites on ISL to form stable adducts. However, ISL exhibited weaker MGO-trapping efficacy compared to the most frequently reported MGO capture agent, phloretin [48]. This was because the trihydroxybenzene group on the A-ring had higher MGO-trapping reactivity than the dihydroxybenzene group, and the limited number of hydroxyl groups on the A-ring restricted the ability of ISL to trap MGO. In summary, ISL exerted anti-glycation activity by trapping MGO.

**Figure 10:**
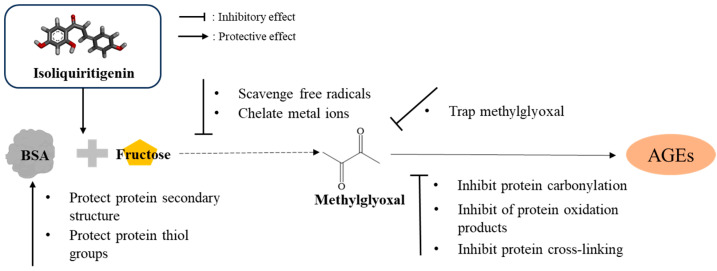
Schematic diagram of the inhibitory mechanism of ISL on BSA glycation.

Based on the above experimental results, ISL exerted its inhibitory effect on BSA glycation through multiple pathways (Figure 10), including inhibiting AGE formation, scavenging free radicals, chelating metal ions, trapping α-dicarbonyl compounds and stabilizing the secondary structure of BSA.

## 4. Conclusions

This study investigated the inhibitory mechanism of ISL on protein non-enzymatic glycation and compared the anti-glycation abilities of ISL in different environments. ISL effectively scavenged free radicals and chelated metal ions, thereby significantly inhibiting AGEs. Meanwhile, ISL effectively protected BSA thiol groups from oxidative modification, reducing protein carbonylation and the content of oxidation products. During the glycation reaction, ISL effectively inhibited glycation modification and protein aggregation by occupying arginine residues and stabilizing the secondary structure of BSA. In addition, ISL can effectively trap MGO, preventing its binding to proteins. In the PEG-simulated crowded environment, the inhibitory effect of ISL on AGEs (29.46%) was weaker compared to that in the PBS system (37.78%), but higher than that of AG in the crowded environment (13.49%). This could be attributed to the crowded environment accelerating the glycation process of BSA. Additionally, the excluded volume effect and increased system viscosity may have limited the binding efficiency of ISL to BSA, thereby reducing the inhibitory effect of ISL on the glycation reaction. Evaluating the anti-glycation activity of ISL under molecular crowding conditions that mimic physiological environments helps more accurately predict its practical application potential in preventing and treating diabetic complications. This study provides new insights into the inhibitory mechanism of ISL on the formation of AGEs and establishes a theoretical foundation for its development as a functional food ingredient for the prevention of diabetic complications.

In the future, nano-encapsulation technology can be employed to improve the penetration, stability and bioavailability of ISL in complex environments. Combined with in vivo models to thoroughly evaluate its efficacy and safety, this approach will facilitate the anti-glycation effects of ISL toward commercial applications. Future efforts could focus on optimizing extraction and purification processes of ISL to improve yield and reduce cost, thereby making it suitable for use as a dietary supplement. Alternatively, ISL-rich plant extracts could be employed as a more cost-effective substitute. Notably, the macromolecular crowding system employed in this study is only a simplified simulation of the real intracellular microenvironment and cannot fully reflect the complex in vivo situation. In addition, the limitations of ISL itself, such as poor water solubility, remain key challenges that urgently need to be overcome in its product development.

## Figures and Tables

**Table 1:** The *K*_sv,_ *K*_a_, *K*_q_ and *n* for the interaction between ISL and BSA in the absence and presence of PEG.

System	*K*_sv_ (×10^5^ L mol^−1^)	*K*_a_ (×10^4^ L mol^−1^)	*K*_q_ (×10^13^ L mol^−1^ s^−1^)	*n*
No Crowding	1.45 ± 0.005	18.89 ± 0.54	1.45 ± 0.005	0.94
PEG	0.50 ± 0.001	6.96 ± 0.48	0.50 ± 0.001	1.27

## Data Availability

The original contributions presented in this study are included in the article. Further inquiries can be directed to the corresponding author.
